# VIEWS OF AGING OF PATIENTS AND SPOUSAL CAREGIVERS PREDICT HOME-BASED REHABILITATION OUTCOMES

**DOI:** 10.1093/geroni/igae098.0549

**Published:** 2024-12-31

**Authors:** Amit Shrira, Irit Shafir

**Affiliations:** Bar-Ilan University, Ramat Gan, HaMerkaz, Israel; Bar Ilan University, Ramat Gan, HaMerkaz, Israel

## Abstract

Home-based hospitalization programs providing health services in patients’ home become increasingly widespread in view of population aging and insufficiencies of inpatient services. Home care has a potential to improve health outcomes and patient satisfaction as well as reduce costs. An impressive body of evidence shows that favorable subjective views of aging (VoA) predict better health outcomes among older adults. Much less is known about the role of VoA in the unique context of home-based hospitalization where care recipients and caregivers are jointly involved in intensive recovery efforts. Therefore, the current study assessed functional outcomes as predicted by VoA of 86 patients (mean age=71.5) and spousal caregivers (mean age=70.5). Patients and spouses receiving post-fracture home-based rehabilitation via one of the largest health maintenance organizations in Israel rated their VoA at the beginning of rehabilitation. Patients’ functional independence was assessed by healthcare personnel at the beginning and end of rehabilitation (approximately two weeks later). The main outcome was rehabilitation effectiveness reflecting the ratio between the absolute functional gain and the patient’s specific potential for functional improvement. Results showed that patients who felt younger or who were less aware of age-related losses had higher rehabilitation effectiveness. Moreover, patients whose spousal caregivers reported less ageist attitudes had higher rehabilitation effectiveness. The study found unique effects of patients’ and spousal caregivers’ VoA on rehabilitation outcomes. Findings suggest that reduction of negative VoA among patients as well as informal caregivers should be considered when tailoring orthopedic rehabilitation frameworks.

